# Correlation between the presence of a cecal appendix and reduced diarrhea severity in primates: new insights into the presumed function of the appendix

**DOI:** 10.1038/s41598-023-43070-5

**Published:** 2023-09-23

**Authors:** Maxime K. Collard, Jérémie Bardin, Bertille Marquet, Michel Laurin, Éric Ogier-Denis

**Affiliations:** 1 Centre de Recherche sur l’Inflammation, INSERM, U1149, CNRS, ERL8252, Team Gut Inflammation, Université de Paris, BP 416, 75018 Paris, France; 2 Department of Colorectal Surgery, Saint-Antoine Hospital, 184 Rue du Faubourg Saint-Antoine, 75012 Paris, France; 3 CR2P (Centre de Recherche en Paléontologie - Paris; UMR 7207), CNRS/MNHN/Sorbonne Université, Muséum National d’Histoire Naturelle, Paris, France; 4 La Vallée des Singes, Zoological Park, Le Gureau, France; 5 INSERM U1242, Centre Eugène Marquis, Université de Rennes 1, Rennes, France

**Keywords:** Gastrointestinal system, Gastrointestinal system

## Abstract

Increased severity or recurrence risk of some specific infectious diarrhea, such a salmonellosis or *Clostridium difficile* colitis, have been reported after an appendectomy in human patients. While several other mammals also possess an appendix, the suspected protective function against diarrhea conferred by this structure is known only in humans. From a retrospective collection of veterinary records of 1251 primates attributed to 45 species, including 13 species with an appendix and 32 without, we identified 2855 episodes of diarrhea, 13% of which were classified as severe diarrhea requiring a therapeutic medication or associated with a fatal issue. We identified a lower risk of severe diarrhea among primate species with an appendix, especially in the early part of life when the risk of diarrhea is maximal. Moreover, we observed a delayed onset of diarrhea and of severe diarrhea in species possessing an appendix. Interestingly, none of the primates with an appendix were diagnosed, treated or died of an acute appendicitis during the 20 years of veterinarian follow-up. These results clarify the function of the appendix among primates, as protection against diarrhea. This supports its presumed function in humans and is congruent with the existence of a selective advantage conferred by this structure.

## Introduction

The cecal appendix, more often called the appendix, is a tight cylindrical structure of the bowel, connected to the inferior margin of the cecum on the proximal end and blind on the tip. The historical hypothesis that this structure is a useless vestige has been refuted by recent studies in the field of evolutionary biology, reporting multiple convergent appearances and very few losses^1–4^. Species possessing an appendix are widely distributed among mammals within various clades such as *Euarchontoglires*, *Monotremata*, *Marsupialia,* and *Afrotheria*^4^. This convergent evolution suggests a selective advantage conferred by the appendix, and hence, a function. The positive correlation between the presence of an appendix and increased longevity in mammals strengthens these conclusions^4^. One of the plausible appendicular functions is that of the colonic microbiota shelter^5^: its shape helps to protect the microbiota contained in the appendix by segregating it from the fecal flow, thus preventing pathogenic dysbiosis caused by infectious diarrhea. In this situation, this healthy microbiota safeguarded in the appendix could recolonise the colon, leading to a faster recovery of the functional colonic microbiota. This hypothesis was triggered by observations of patients with a history of appendectomy: a higher risk of diarrhea recurrence in case of *Clostridium difficile* infection, a higher risk of diarrhea due to a non-typhoidal *Salmonella* infection, and also a higher risk of hospitalization in patients infected by this bacterium^6,7^. Nevertheless, the role of the appendix in preventing severe diarrheal disease is complex to explore in humans for several reasons. Namely, the antibiotics used to treat diarrheal disease vary over time and between institutions, and the controls with an appendix vary among studies. Additionally, the direct causal link between appendectomy and an increased risk of infectious diarrhea has never been conclusively demonstrated and the statistical association found in studies^6,7^ could be explained by a mechanism other than the colonic microbiota reservoir of the appendix. This hypothetical function of appendicular microbiota safe-house is currently supported only by these observations in humans; no evidence from other mammal species has been reported yet. Primates constitute a clade of interest to test this hypothesis because the appendix appeared several times in this clade^4^; thus some primate species have an appendix, while other species do not. Additionally, a high incidence of diarrhea is reported among primates^8^. The aim of this study is to compare the frequency and severity of diarrhea episodes between primate species with and without appendix to investigate the function of the appendix in primates based on its suspected function in humans.

## Results

### Frequency and severity of diarrhea episodes

**Figure 1:**
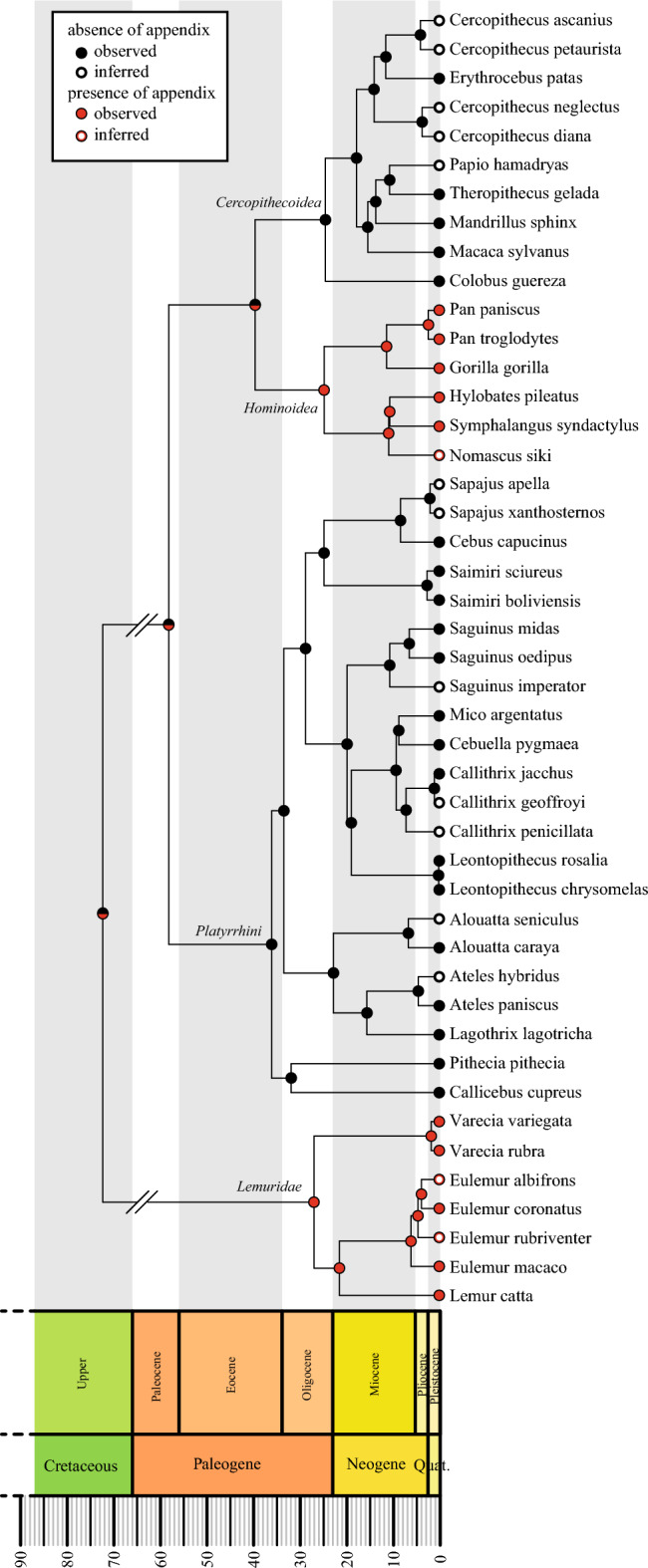
Taxonomic distribution of the appendix in studied primate species. Subset of the phylogeny (tree n° 1 node-dated). Colors correspond to absence (black) and presence (red) of appendix. Solid circles refer to observed appendix presence or absence; empty circles correspond to inferred appendix presence or absence.

We assessed the frequency, the severity and the timing of diarrhea in a cohort of 1251 captive primates belonging to 45 species, along with a possible influence of the presence of an appendix. Our cohort included all primate residents of the zoological park “La Vallée des Singes” (Romagne, France) between January 1998 and December 2018. Forty-nine percent of the individuals of this cohort had at least one episode of diarrhea documented in the medical database Species360 ZIMS (Zoological Information Management Software). In total, 2855 episodes of diarrhea were diagnosed during the follow-up period and 13% (371 episodes) were classified as severe. Twenty-five animals died within 14 days following the diagnosis of their last episode of diarrhea. The presence or absence of an appendix was known from published observations of anatomical dissections for 30 of the 45 species, including 151 individuals belonging to 10 species possessing an appendix and 888 individuals belonging to 20 species without appendix. Among the 15 species without published information about presence or absence of the appendix, the appropriate state was inferred using the parsimony criterion, which indicated that 3 had “inferred presence” and 12 had “inferred absence”^3^. Finally, the entire cohort of 45 species (including those with inferred presence) included 172 individuals of 13 species with appendix and 1079 individuals of 32 species without appendix. The presence or absence of appendix among the 45 species was heterogeneously distributed within 4 clades: all species in *Strepsirrhini* (represented here exclusively by the *Lemuridae*) and *Hominoidea* possessed an appendix, whereas all species in *Platyrrhini* and *Cercopithecoidea* lacked an appendix (Fig. 1). Note that this distribution is contingent on our sample; other species from these 4 clades and not present in our cohort can display variability in this character^3,4^.

### Influence of the presence of an appendix on diarrhea frequency

**Figure 2:**
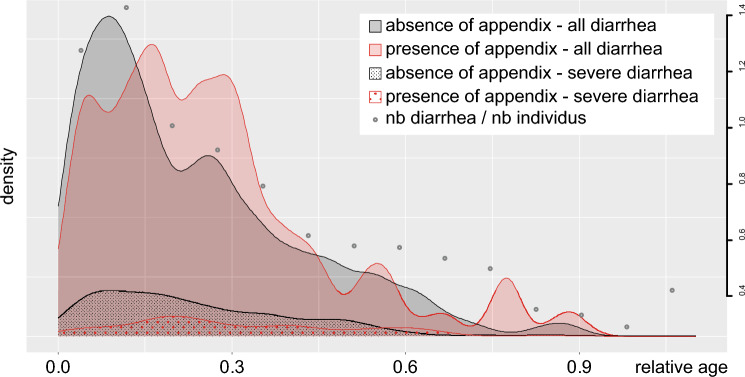
Incidence of diarrhea and severe diarrhea over the lifetime of primates with and without an appendix in the entire cohort. Appendix bearers experience their peak frequency of diarrhea later in life and suffer from fewer severe diarrhea episodes. The four curves represent the probability distributions (kernel density estimates) of the relative ages of diarrhea occurrences for individuals with an appendix (red) or without (black) and highlighting the severe diarrhea (dotted pattern; full color pattern includes all diarrhea). Diarrhea episodes have been equally sampled in individuals with, or without an appendix, regardless of their species. Ages have been standardized using average maximum longevity of each of the 45 species. Grey dots represent the number (nb) of diarrhea episodes divided by the number of individuals that have been observed in each time bin (right y-axis). This variable shows that the decreasing quantity of diarrhea throughout life is a genuine pattern.

We found that diarrhea occurred most frequently during the first quarter of life, decreasing progressively thereafter (Fig. 2 for all species and Supplementary Fig. S1 without inferred presence/absence). To account for phylogenetic effects, bootstrapping was performed by sampling separately the individuals in the four aforementioned clades (Fig. 1). The frequency of diarrhea episodes standardised by period of observation was lower in primates with an appendix than in primates without an appendix in 85.1% of bootstrap iterations considering all individuals (*p* = 0.149) and in 83.9% after exclusion of inferred presence (*p* = 0.161). This pattern was much sharper when only severe diarrhea were considered, as 99.9999% of the iterations revealed a lower number of severe diarrhea in primates with appendix than those without appendix (*p* < 0.0001), whether or not the inferred presence/absence was included (Supplementary Table S1).

### Impact of the presence of an appendix on the timing of diarrhea occurrence

We then assessed if the presence of the appendix changed the timing of diarrhea occurrence during the life. Median age of diarrhea occurrence was significantly delayed in the primates possessing an appendix (median age standardised on the maximal observed longevity with *versus* without appendix: 0.2355666 versus 0.194863, Wilcoxon test *p* < 0.0001 considering the entire cohort and 0.2306849 versus 0.1956251, Wilcoxon test *p* < 0.0001 after exclusion of inferred presence/absence). This delayed onset of diarrhea in primates with an appendix was also observed when only severe diarrhea episodes were considered (median age ratio value standardised on the maximal observed longevity with versus without appendix: 0.2403487 versus 0.2068493, Wilcoxon test *p* < 0.0001 considering the entire cohort and 0.26132 versus 0.181154, Wilcoxon test *p* = 0.0016 after exclusion of inferred presence).

**Figure 3:**
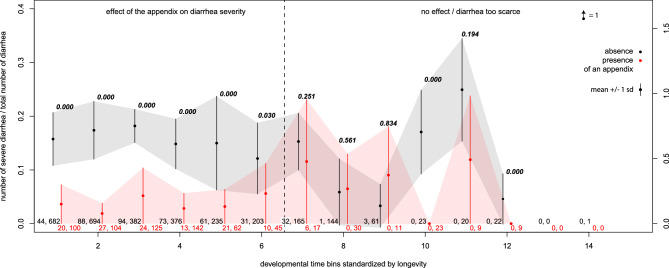
Proportion of severe diarrhea among all diarrhea episodes of the entire cohort. Diarrhea episodes are less severe for individuals with an appendix during the first third of their life; after, the effect disappears and/or diarrhea are too scarce to quantify the effect. 1000 bootstrap sampling were performed in each of the four clades, then respectively averaged in clades without (*Cercopithecoidea* and *Platyrrhini*) and with (*Lemuridae* and *Hominoidea*) an appendix (Fig. 1). Dots and bars correspond to the mean and two standard deviations of the generated distributions (± 1 sd). This procedure was repeated in 14 time bins standardized by the maximal recorded longevity of each of the 45 species in our dataset. Numbers below correspond to the number of diarrheas in each of the four clades. Values above are *p*-values corresponding to the fraction of the bootstrap runs in which proportion of severe diarrhea is at least as high for individuals with an appendix as for individuals without an appendix. In the 14th time bin, an outlier (only one diarrhea recorded) has been moved to enhance legibility.

If the standardised lifetime is arbitrarily split into 14 time bins of equal duration, the proportion of severe diarrhea among all diarrhea episodes is significantly lower in primates possessing an appendix during the first six time bins (Fig. 3 for all individuals). These 6 time bins coincide with the peak of diarrhea frequency observed in the cohort. After these initial 6 periods, while the diarrhea frequency is considerably lower, the proportion of severe diarrhea among all diarrhea episodes no longer differs between the two groups. A similar pattern is observed after exclusion of inferred presence (Supplementary Fig. S2).

### Risk of appendicitis among primates possessing an appendix

Within the 172 primates of our cohort with appendix, none of them was diagnosed or treated for an acute appendicitis. Thirty-two of them passed away between 1998 and 2018. No acute appendicitis was found during systematic autopsies.

## Discussion

In summary, presence of an appendix is significantly correlated with a reduced severity and a delayed occurrence of diarrhea episodes in a cohort of 45 species of primates. One possible mechanism by which the appendix may protect against severe diarrhea is by sheltering the microbiota. A study conducted on the aye-aye (*Daubentonia madagascariensis)* revealed a different composition of the endo-appendicular microbiota from the rest of the colonic microbiota, thereby supporting this concept of appendicular microbiota safe-house^9^. The exclusion of the endo-appendicular microbiota from the fecal flow^10^ may facilitate the re-inoculation of the healthy microbiota into the colon during an infectious diarrhea^11^. Evidence in studies on human populations supporting the safe-house or re-inoculation model of the appendix function^6,7^, in combination with our observations from other primates, indicate that the ratio of severe/non severe diarrheal disease is significantly lowered, plausibly because of a more resilient bowel microbiota conferred by the presence of an appendix. However, this suspected mechanism to explain the statistical association we observe between the presence of an appendix and the reduction in diarrhea severity cannot be definitively established from our observational data, especially considering the coexistence of other hypotheses. An alternative hypothesis reflects the fact that the appendix is a lymphoid organ involved in the immune system education. In humans, the appendix is known to be rich in M cells^12^, ensuring a trancytosis from the appendicular lumen towards antigen-presenting cells that can then interact with B and T lymphocytes present in high quantities in the appendix within the lymphoid follicles^13^. This immune priming in the appendix could reduce the severity of infectious diarrhea by enhancing the efficiency of the immune response. It remains to be seen whether the appendix of other primates shows these same histological characteristics. In our cohort, diarrhea and severe diarrhea were more frequent at the beginning of life and progressively declined afterwards. This distribution could be explained by the acquisition of an immunity against enteric pathogens during the first part of life, although this hypothesis, already proposed in humans^14^, has never been documented in other primates. We show that the appendix affects diarrhea severity before the end of the first half of life, which is concomitant with the peak of diarrhea and severe diarrhea incidence. Interestingly, the loss of this function by aging is also concomitant with the involution of the appendix characterised by the progressive atrophy of its lymphoid structures and loss of immune function^13^.

Whatever the exact function of the appendix, the reduction in the severity of diarrhea that we observe constitutes a selective advantage that may explain the evolutionary history of this structure among primates. In our cohort of 1251 primates, only 25 individuals died within 14 days following the diagnosis of a diarrhea over the study period. This mortality associated with a diarrhea episode is not representative of the diarrhea mortality in wild primates because the veterinary treatment, notably through antibiotic therapy, antidiarrheal drugs and rehydration, greatly reduces diarrhea-induced mortality, too far below the 11% of mortality per diarrhea episode in the various primate species reported in the literature for wild populations^8^. In our previous study, we discovered that the presence of an appendix is correlated with a longer maximum longevity among mammals possessing it^4^. Considering William’s theory that predicts an increased maximal longevity in species when extrinsic mortality is reduced^15^, our previous results suggest that the appendix should reduce extrinsic mortality of mammals possessing it, and our new results about the risk of severe diarrhea support this hypothesis. We also found that the appendix is associated with a delay in the age of onset of diarrhea, especially of severe diarrhea, so conferring the protection against severe diarrhea in the early part of life, when the reproductive potential is maximal^16,17^. This benefit should positively influence reproduction, providing a substantial selective advantage for the survival of these individuals.

Our analyses with inferred presence/absence, have a limitation that should be mentioned. Some species have been inferred regarding the presence or absence of an appendix despite a reported polymorphism in the literature^18^ (5 out of the 15 inferred species), meaning that a discrepancy exists between different anatomical reports regarding the presence or absence of an appendix in the same species. This suspicion of polymorphism involves only 20 individuals in our cohort of 1251 primates. The polymorphism may be more restricted, as in some cases, interpretation of a structure as an anatomical appendix can also vary among observers, especially in borderline anatomical cases, and may also depend on the quality of the studied specimens. Fisher^18^ declared, about possible polymorphism of 5 primate species that we sampled: “A review of the literature reveals that the confusion arises because several different, and sometimes contradictory, criteria are enlisted to distinguish an appendix.” Additionally, age-related variations, including a potential involution of the appendix with aging, might mistakenly lead to the conclusion of appendix polymorphism within a species. Fisher has also clearly identified this potential bias in the definition of appendicular polymorphism: “As most publications fail to report the age of the primate specimens, it is difficult to rule out ontogenetic shape change in producing some degree of the observed variation. Finally, cecal shape can be altered dramatically by various preservation techniques”^18^. So, we opted to resolve as best we could, given all the data at hand, cases of possible polymorphism to score what appears to be the most plausible state. The advantage of this approach is that species that may be genuinely polymorphic, but in which one of the two states is present in a fairly strong majority of individuals, remain informative for our analyses.

What about the function of the appendix in humans? In 2015, diarrhea mortality was identified as the second leading cause of death in children between 1 month and 5 years of age^19^, along with some infectious diarrhea, such as cholera^20^, which continue to produce epidemics that generate high mortality rates in the absence of appropriate medical treatment. If the function of the appendix in humans is the same as in other primates, the protection conferred by the appendix against diarrhea more than offsets the risk of developing fatal appendicitis. Interestingly, none of the primates of our cohort with appendix was diagnosed or treated for acute appendicitis. Although some exceptional cases of appendicitis in non-human primates have been authenticated in the literature^21,22^, its frequency is much lower than in humans, for whom the lifetime risk of appendicitis is estimated to be between 9 and 16%^23,24^. An increased risk of appendicitis related to the lifestyle of humans in industralised countries could explain this discrepancy between humans and other primates^25,26^. In addition, recent studies highlighted that in humans, a substantial proportion of mildly inflamed appendicitis may be spontaneously reversible without any antibiotics or appendectomies^27^, questioning a possible overdiagnosis of certain physiological appendicular inflammations due to the performance and accessibility of modern medical imaging^28^.

## Methods

### Population of non-human primates

We reviewed retrospectively all the medical records from all primate residents between January 1998 and December 2018 in the zoological park “La Vallée des Singes” (Romagne, France). In this park dedicated to primates, animals are in semi-freedom on wooded islands separated from each other by impassable obstacles (water or fences). Animal caretakers and veterinarians wear new gowns and overshoes each time they move between monkey territories to avoid spreading germs between islands. Feeding of each animal is qualitatively and quantitatively adapted to its species. The meat is systematically cooked, the vegetables are always washed and the croquettes are industrially manufactured at high temperature. Each animal is clinically observed at least twice a day by a caretaker, once in the morning and once in the afternoon. In case of any abnormalities noticed during these clinical examinations, a veterinarian specialised in primates is immediately contacted by the caretaker to discuss the appropriate measures to be taken. In addition, all anomalies observed during the clinical examination and all treatments given are reported prospectively in a computerised database called the Species360 ZIMS (Zoological Information Management Software). Finally, all animals that die in the zoo undergo a systematic autopsy to assess the cause of the death, whatever the clinical context.

From the Species360 ZIMS database, we obtained the identification number of all primates that were housed at least one day in the zoo between 1998 and 2018. For each animal, we collected data on its species, sex, date of birth, date of arrival in the zoo, date of death or date of departure from the zoo if applicable. In addition, we extracted from the Species360 ZIMS database all the medical observations written by the caretakers or veterinarians mentioning the word "diarrhea" during the period of the study. The date of each of these observations was also collected. Then, all of these computerised observations were re-read to confirm the diagnosis of diarrhea written in the observations. Two observations of diarrhea for the same individual occurring no more than 14 days from each other were considered to be the same episode of diarrhea. Each episode of diarrhea was classified as severe or non-severe. A non-severe diarrhea means that the veterinarian decided that any medical treatment was not justified given the clinical tolerance to diarrhea of the animal and that the primate did not pass away within 14 days following the diagnosis of diarrhea.

### Information related to species

Our definition of the appendix is the same as in previous comparative studies^3,4^. The appendix was defined morphologically as a close-ended projection that is clearly differentiated from the cecum or the colon by a change in diameter, whatever the density of lymphoid follicles in this structure. From the open-access database of our previous publication^4^, we extracted the information about the presence or absence of the appendix for the following species: *Aouatta seniculus, Callicebus cupreus, Cebuella pygmaea, Colobus guereza, Erythrocebus patas, Eulemur coronatus, Eulemur macaco, Gorilla gorilla, Lagothrix lagotricha, Lemur catta, Pan troglodytes, Pithecia pithecia, Saguinus midas, Theropithecus gelada* and *Varecia variegata*. From the publication of McGrosky et al.^29^, two species were corrected in our database, given that pictures clearly show that *Callithrix jacchus* and *Mico argentatus* lack an appendix. Moreover, from the same publication^29^, we collected information about the presence or absence of appendix for *Ateles paniscus, Leontopithecus chrysomelas, Macaca sylvanus, Mandrillus sphinx, Saguinus oedipus, Saimiri boliviensis, Saimiri sciureus, Symphalangus syndactylus* and *Varecia rubra*. Information about the appendix was obtained for *Cebus capucinus, Hylobates pileatus* and *Leontopithecus rosalia* from additional studies^30–32^. A picture of the caecal region taken during the autopsy of an individual that died in zoological park “La Vallée des Singes” reveals the presence an appendix in *Pan paniscus* (Supplementary Fig. S3).

The literature yielded unambiguous data about the presence or absence of an appendix for 30 of the 45 species present in our database (10 with an appendix and 20 without); these data were called “observed appendix”. Additionally, we conducted a parsimony optimization with the 15 species without information in the literature about the appendix (*Nomascus siki, Sapajus apella, Sapajus xanthosternos, Saguinus imperator, Callithrix geoffroyi, Callithrix penicillata, Alouatta seniculus, Ateles hybridus, Eulemur albifrons and Eulemur rubriventer*) or with a suspicion of polymorphism defined by discrepancies among anatomical reports regarding the presence or absence of an appendix in a given species (*Cercopithecus ascanius, Cercopithecus petaurista, Cercopithecus diana, Cercopithecus neglectus, Papio hamadryas*)^18^. These inferences was performed without encountering any case of optimization ambiguity for any of the 15 species.

We collected the maximal longevity observed for all 45 primate species of our database from two databases available online: the database Animal Diversity Web (ADW) (https://animaldiversity.org/) and the database AnAge (http://genomics.senescence.info/species/). When these two databases provided different maximum observed longevities for the same species, the longest longevity was kept. The timing of occurrence of all diarrhea episodes was standardized by dividing the age at which an individual experienced that episode by the maximal longevity recorded for its species.

As some uncertainties persist about the evolutionary history of primates, we considered two sets of 100 Bayesian time-trees (one of node-dated trees and another one of tip-dated trees) elaborated by Upham et al.^33^ and available on https://vertlife.org/. These trees were sampled from the “DNA-only” phylogenetic trees and filtered to retain only the 45 different species of primates present in our database. We used these two sets of phylogenetic trees to assess the impact of phylogenetic uncertainty on our statistical analyses.

### Effect of appendix on the diarrhea frequency

To test the hypothesis that the appendix affects the frequency of diarrhea, we consider the difference between the frequencies of diarrhea episodes per individual, separating those without and with an appendix. These frequencies are calculated as the average number of episodes per individual divided by the period of observation of each individual. If the frequency difference between individuals without and with appendix is positive, the appendix has a beneficial effect. We refine our estimation by performing bootstrap resampling to get closer to the sampling distribution of this variable and to take into account possible bias due to unequal individual sample size among species or clades. This approach has been performed for every combination of total number of diarrhea/only severe diarrhea; observed/inferred presence of appendices; sampling in species/clades.

Our approach of bootstrap sampling of individuals or diarrhea in the four clades (Fig. 1) has two benefits: (1) taking into account the distribution of the variable in each clade and (2) giving an equal weight to each clade to correct bias of overrepresented clades. The algorithm works as follows: (1) take each clade, (2) resample the individuals or diarrhea with replacement, (3) compute the difference between frequencies of diarrhea episodes in clades without appendix and clades with an appendix, (4) redo step 1–3 until results stabilize (in our case 1000 was enough). The resulting distribution of the differences can be used to test the hypothesis of the appendix effect on diarrhea frequencies. Counting the number of times where this difference of frequencies is negative (i.e. frequency of diarrhea with appendix is higher) or nil can be interpreted as the *p*-value of the null hypothesis that the frequency of diarrhea in individuals with an appendix is higher than or equivalent to the frequency in individuals without an appendix. Clades within which resampling was performed have been chosen based on the appendix ancestral state reconstruction. The 200 trees selected show four relevant clades; for two of them, *Strepsirrhini* (including exclusively species of the *Lemuridae*) and *Hominoidea* include only species with an appendix in our sample, and the other two clades for which our sample included only species without appendix, namely *Platyrrhini* and *Cercopithecoidea* (Fig. 1)). This distribution ensures equal weighting of the presence and absence of appendix and it controls for phylogenetic effects (as if we had only four statistically independent individuals: the four clades). All trees show exactly the same distribution of the appendix into these 4 clades, and our results were always exactly the same on all trees. Therefore, only one tree has been used for the analyses.

### Occurrence of diarrhea during lifespan

The distribution of the occurrences of diarrhea in the life of all individuals was obtained by dividing the age at which each episode occurred by the estimated maximum longevity of their species. We also sampled with replacement the same number of individuals with or without appendix to standardize the distributions. The resulting distributions for all diarrhea/only severe diarrhea and absence or presence of appendix are unimodal and the mode for individuals with appendix seems to be at a higher relative age (Fig. 2). To statistically test this interpretation, we performed a series of Wilcoxon tests on the relative age values between the individuals with or without an appendix. Tests on all combinations of all diarrhea/only severe ones and inferred or observed appendix presence have all been calculated.

### Severity of diarrhea during lifespan

**Figure 4:**
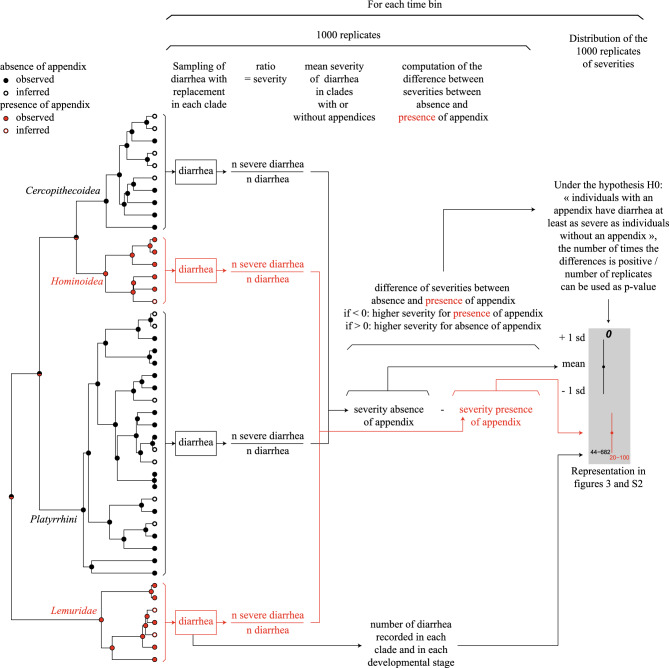
Flowchart representing the successive stages of the analysis in bootstrap sampling of diarrhea in the four clades to compare the severity of diarrhea over the lifespan of primates with and without appendix.

As both the severity of diarrhea and its occurrence in individual’s life seem to be affected by the absence or the presence of an appendix, we performed an integrative analysis including all these variables. It involves splitting the relative lifespan of individuals in several time bins. In each time bin, we sampled with replacement diarrhea episodes in the four above-mentioned clades. We then computed the ratio between the number of severe over the total number of diarrhea in each clade and then averaged these values in clades having an appendix and in those without appendix. By repeating the procedure a large number of times, we obtained an estimate of the distributions of this ratio for the two sets characterized by the absence or the presence of the appendix. In each run, we also computed the difference between average ratios of the two sets serving as the basis to compute the p-value corresponding to the null hypothesis that individuals with an appendix have diarrhea at least as severe as individuals without an appendix. This method is summarized in Fig. 4 and was used to produce Fig. 3 for all species and Supplementary Fig. S2 for species with observed appendix only.

### Supplementary Information

Supplementary Information.

## Data Availability

The datasets used and analyzed during the current study are available from the corresponding author on reasonable request.
