# Supplementary material for: Correlation between the presence of a cecal appendix and reduced diarrhea severity in primates: new insights into the presumed function of the appendix
Source: Sci Rep. 2023 Sep 23;13:15897. doi: 10.1038/s41598-023-43070-5 (PMC10517977; doi:10.1038/s41598-023-43070-5)
Supplement: Supplementary file 1 — Supplementary Information. [file 41598_2023_43070_MOESM1_ESM.docx]

**Supplementary information**

The cecal appendix protects against severe diarrhea in primates

*Maxime K. Collard, Jérémie Bardin^,^, Bertille Marquet, Michel Laurin, Éric Ogier-Denis*

**This file includes:**

Supplementary figures S1 to S3

Supplementary table S1

**
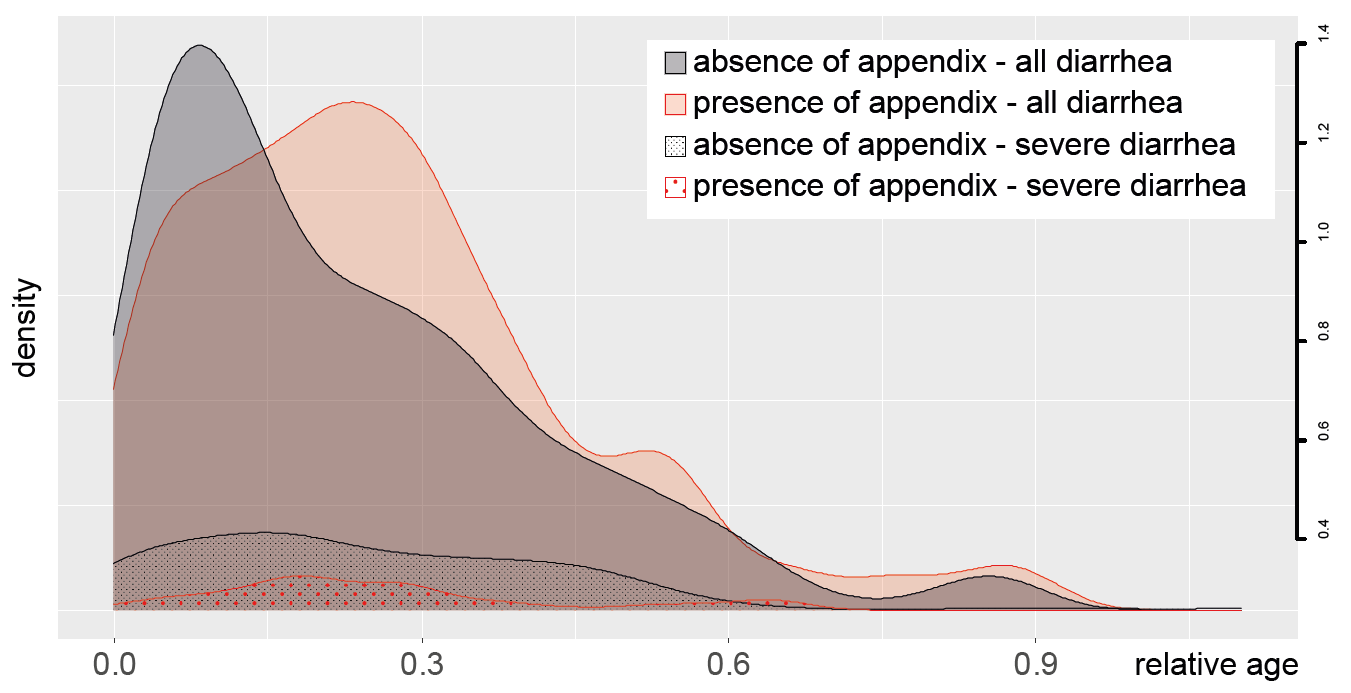
**

**Supplementary** **Fig. S1. Frequency of diarrhea and only severe diarrhea over ontogenetic time in primates with and without appendix in the studied cohort after exclusion of species with inferred appendix presence.**

The four curves represents the probability distributions (kernel density estimates) of the relative ages of diarrhea occurrences for individuals with (red), or without (black) an appendix and highlighting the severe diarrhea (dotted pattern; full color pattern includes all diarrhea). Diarrhea episodes have been equally sampled in individuals with, or without an appendix, regardless of their species. Ages have been standardized using maximum longevity of each of the 30 species (without inferred presence).

**
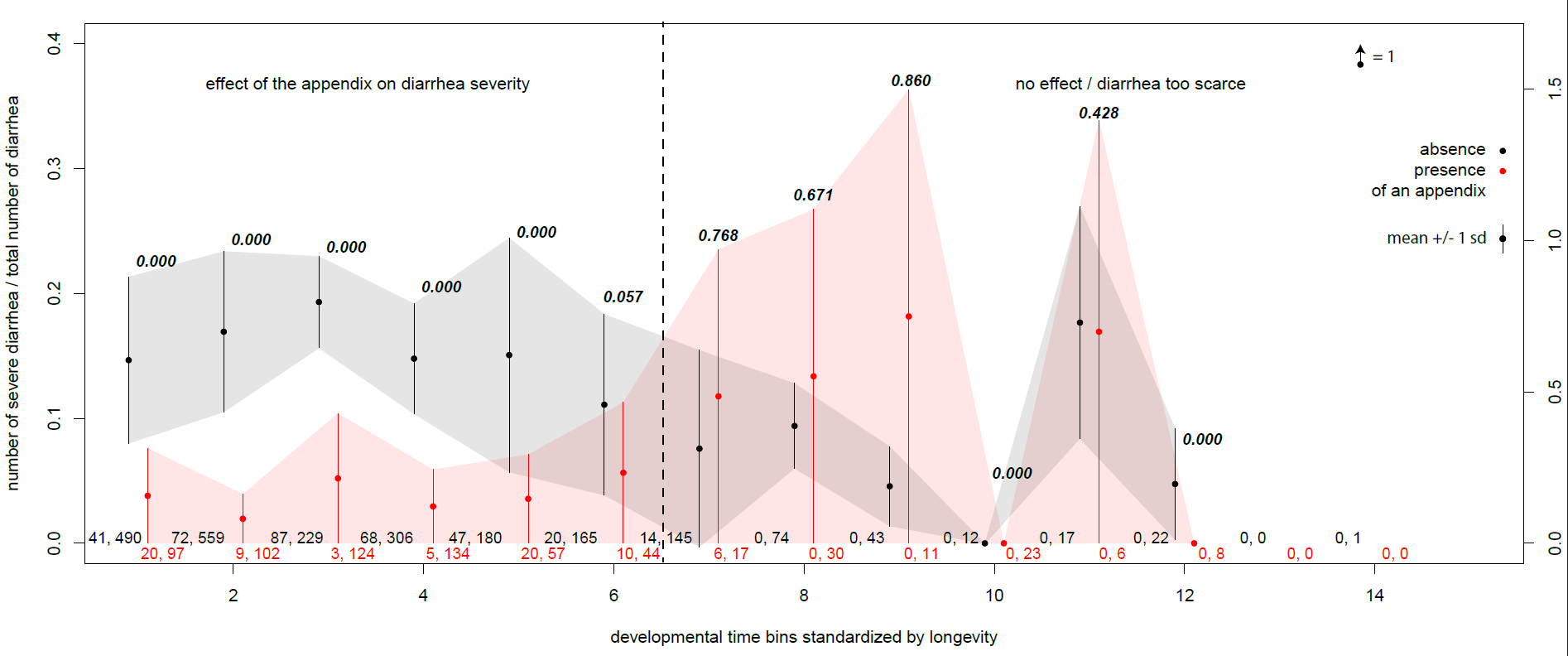
**

**Supplementary** **Fig. S2. Proportion of severe diarrhea among all diarrhea episodes over the lifetime (species with inferred appendix presence or absence excluded).**

Diarrhea is less severe for individuals with an appendix during the first third of their life; after, the effect disappears and/or diarrhea are too scarce to quantify the effect. 1000 bootstrap samples have been obtained in each of the four clades, then respectively averaged in clades without (*Cercopithecoidea* and *Platyrrhini*) and with (*Lemuridae* and *Hominoidea*) an appendix (Fig. 1). Dots and bars correspond to the mean and two standard deviations of the generated distributions (+/- 1 sd). This procedure has been repeated in 14 time bins standardized by the maximal recorded longevity of each of the 30 species (without inferred presence) in our dataset. Numbers below correspond to the number of diarrheas in each of the four clades. Values above are p-values corresponding to the fraction of the bootstrap runs in which proportion of severe diarrhea is at least as high for individuals with an appendix than for individuals without an appendix. In the 14th time bin, an outlier (only one diarrhea recorded) has been moved to enhance legibility.

**
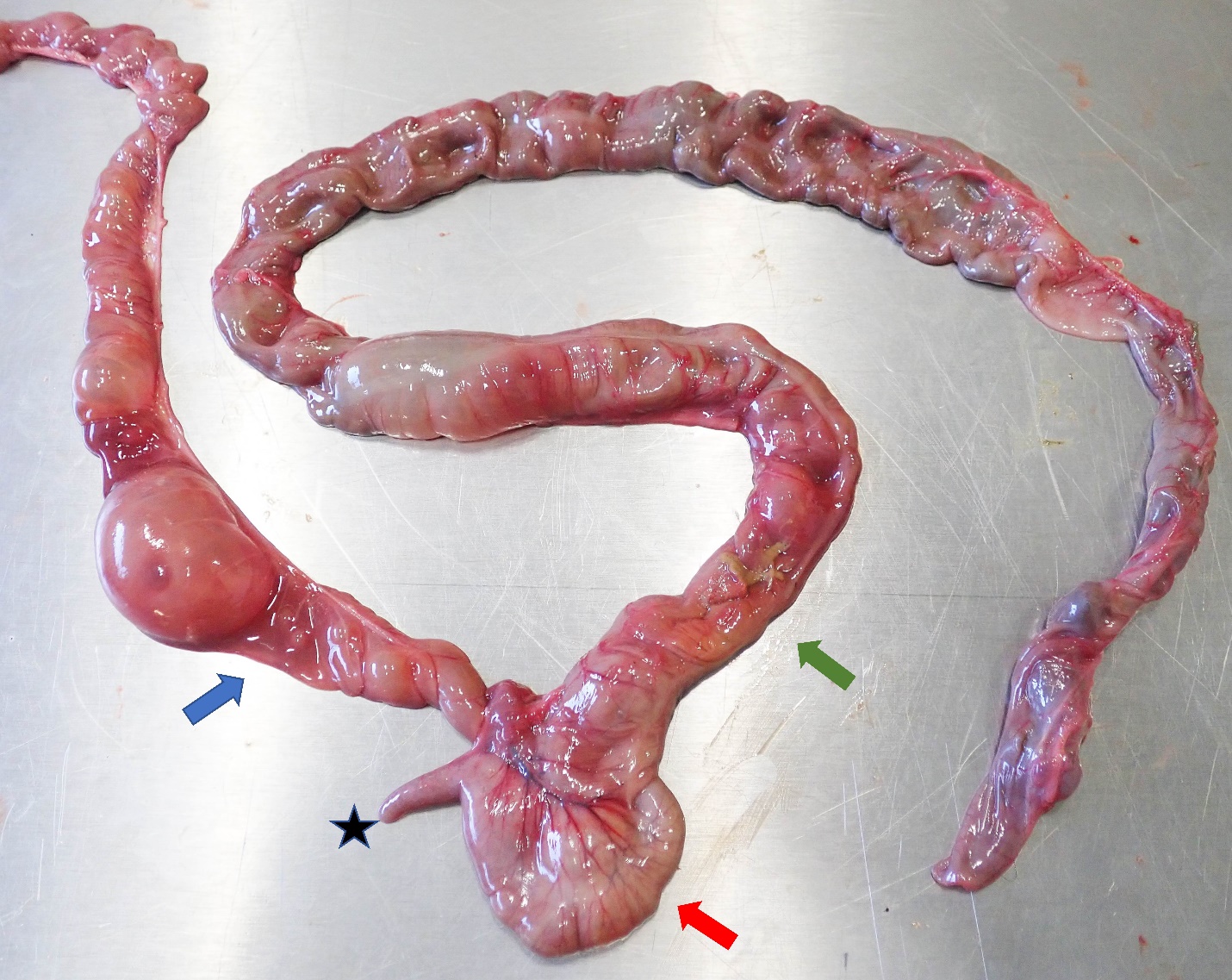
**

**Supplementary** **Fig. S3. Picture of the colon and small bowel from a *Pan paniscus* monkey.**

The presence of an appendix (black star) in *Pan paniscus* is attested by this picture from an a autopsied primate of this species at the zoological park “La Vallée des Singes” (Romagne, France). Terminal ileon, caecum and the rest of the colon are identified by blue, red and green arrows respectively.

**Supplementary** **Table S1. Overview of the distribution of diarrhea episodes primate species with an appendix to those without.**

This table presents the results evoked in the text.

|  | With inferred presence | | | Without inferred absence | | |
| --- | --- | --- | --- | --- | --- | --- |
|  | With appendix | Without appendix | p-value | With appendix | Without appendix | p-value |
| *Number of individuals* | 172 | 1079 | - | 151 | 888 | - |
| *Number of species* | 13 | 32 | - | 10 | 20 | - |
| *Proportion of bootstrap iterations with lower frequency of diarrhea episodes standardised by period of observation in primates with appendix comparated to primates without* | 85.1% | | 0.149 | 83.9% | | 0.161 |
| *Proportion of bootstrap iterations with lower frequency of severe diarrhea episodes standardised by period of observation in primates with appendix comparated to primates without* | 99.9999% | | <0.0001 | 99.9999% | | <0.0001 |
| *Median age of diarrhea occurrence standardised on the maximal observed longevity* | 0.2355666 | 0.194863 | <0.0001 | 0.2306849 | 0.1956251 | <0.0001 |
| *Median age of severe diarrhea occurrence standardised on the maximal observed longevity* | 0.2403487 | 0.2068493 | <0.0001 | 0.26132 | 0.181154 | 0.0016 |
